# Testing a Human Antimicrobial RNase Chimera Against Bacterial Resistance

**DOI:** 10.3389/fmicb.2019.01357

**Published:** 2019-06-19

**Authors:** Guillem Prats-Ejarque, Jiarui Li, Fatima Ait-Ichou, Helena Lorente, Ester Boix

**Affiliations:** Faculty of Biosciences, Department of Biochemistry and Molecular Biology, Universitat Autònoma de Barcelona, Barcelona, Spain

**Keywords:** RNase, antimicrobial resistance, antibiotic adjuvant, gram-negative bacteria, antimicrobial peptides

## Abstract

The emergence of bacterial resistance to the most commonly used antibiotics encourages the design of novel antimicrobial drugs. Antimicrobial proteins and peptides (AMPs) are the key players in host innate immunity. They exert a rapid and multifaceted action that reduces the development of bacterial adaptation mechanisms. Human antimicrobial RNases belonging to the vertebrate specific RNase A superfamily participate in the maintenance of tissue and body fluid sterility. Among the eight human canonical RNases, RNase 3 stands out as the most cationic and effective bactericidal protein against Gram-negative species. Its enhanced ability to disrupt the bacterial cell wall has evolved in detriment of its catalytic activity. Based on structure-functional studies we have designed an RNase 3/1 hybrid construct that combines the high catalytic activity of RNase 1 with RNase 3 bactericidal properties. Next, we have explored the ability of this hybrid RNase to target the development of bacterial resistance on an *Acinetobacter baumannii* cell culture. Synergy assays were performed in combination with colistin, a standard antimicrobial peptide used as an antibiotic to treat severe infections. Positive synergism was observed between colistin and the RNase 3/1 hybrid protein. Subsequently, using an *in vitro* experimental evolution assay, by exposure of a bacterial culture to colistin at incremental doses, we demonstrated the ability of the RNase 3/1 construct to reduce the emergence of bacterial antimicrobial resistance. The results advance the potential applicability of RNase-based drugs as antibiotic adjuvants.

## Introduction

The emergence of bacterial resistance to conventional antibiotics is becoming a serious sanitary and economical threat. There is an urgent need to develop alternative drugs that address this global health issue and overcome the spread of infectious diseases (Lohrasbi et al., 2018; Mustazzolu et al., 2018). In particular, novel therapeutic approaches will be required to fight the dissemination of multi-drug resistant bacterial strains that threaten our public health system to return to the “pre-antibiotic era” (Wright, 2016). Currently, the majority of antimicrobial agents that are available in the pharmaceutical market rely on the direct elimination of the microorganisms. Notwithstanding, recent literature highlights the potentiality of drug combination and simultaneous targeting of diverse cellular processes (Brochado et al., 2018; Lázár et al., 2018) to overcome the bacterial development of resistance mechanisms. Being aware that full eradication of antibiotic resistance might be unattainable, we should gather all our available means to minimize its dissemination and impact. One of the favorite strategies to overcome the so called “antibiotic resistance era” relies on the discovery of unconventional drugs and combinatorial approaches (Brown and Wright, 2016).

In the search of novel antibiotic candidates, our own innate immunity system represents one of the best reservoirs. Upon infection, our innate immune cells secrete a variety of antimicrobial proteins and peptides (AMPs) that protect the host biological fluids against pathogen invasion. AMPs are usually small polypeptides that present a non-specific wide-spectrum targeting of microorganisms (Hancock and Diamond, 2000). A growing clinical interest for AMPs is derived from their low toxicity to mammalian cells, together with the fact that bacterial resistance to these molecules seems inherently more difficult to acquire in comparison to conventional antibiotics (Lai and Gallo, 2009; Nakatsuji and Gallo, 2012; Casciaro et al., 2018). Within the AMPs, the largest group corresponds to cationic peptides (Hancock and Diamond, 2000). The mode of action of cationic antimicrobial peptides is different from that of conventional antibiotics and is often related to interaction with bacterial walls through electrostatic forces and subsequent cell lysis (Yeaman, 2003; Bahar and Ren, 2013). AMPs are mostly amphiphilic in nature and they are comprised of hydrophobic and hydrophilic residues aligned on opposite sides of the peptides, facilitating their easy penetration through cell membranes (Torrent et al., 2011a,b; Brandenburg et al., 2012). Fortunately, although several cases of bacterial resistance to AMPs have been observed (Perron et al., 2006; Moffatt et al., 2010), the complex structure of bacterial envelopes hinder the development of total resistance. Besides, AMPs are frequently multifaceted molecules that combine a direct mechanical action to the bacterial envelope with a specific enzymatic activity that targets essential intracellular macromolecule components. A variety of multifunctional antimicrobial proteins, endowed with protease, DNase or RNase activity, can participate in the host defense system (Brogden, 2005; Hancock and Sahl, 2006; Boix and Nogués, 2007; Arranz-Trullén et al., 2017).

In our laboratory, we are working on the structure-functional relationship of human antimicrobial RNases that belong to the vertebrate-specific RNase A superfamily and are secreted by epithelial and blood cells during infection (Gupta et al., 2012; Koczera et al., 2016; Lu et al., 2018). Characterization of the mechanism of action of human antimicrobial RNases suggests that a combination of activities are taking place (Salazar et al., 2016; Lu et al., 2018). In particular, the human RNase 3, mostly secreted by eosinophils upon infection, combines a high cationicity (pI ∼11), lipopolysaccharide (LPS) binding affinity, membrane destabilization and bacterial agglutination activities (Boix et al., 2012; Torrent et al., 2012).

Interestingly, a striking structural homology between the RNase A superfamily and bacterial RNases belonging to the contact-dependent growth inhibition (CDI) toxins was recently reported (Batot et al., 2017; Cuthbert et al., 2018). CDI bacterial toxins work as inter-strain competition weapons and use the RNase enzymatic activity as a self-defense mechanism. Indeed, most of our current antibiotics are derived from natural compounds produced by microorganisms against competing species (Blair et al., 2015). Among them, bacteriocins, expressed by commensal species and endowed with enzymatic activities, are being considered as appealing alternative antibiotics to fight pathogenic strains (Mathur et al., 2018).

Another alternative approach proposed to fight resistance mechanisms against antimicrobial agents is the targeting of the bacterial community integrity. Recent studies reveal the previously underestimated complexity of bacterial communities and identify collective resistance mechanisms (Vega and Gore, 2014). Novel strategies can be engineered to target the bacterial community cohesion and thereby weaken collective resistance. Collective resistance ensures the survival of the microbial community upon exposure to antibiotic conditions that otherwise would be lethal to individual bacterial cells (Vega and Gore, 2014). One of the main mechanisms that ensures bacterial survival relies on the intercellular horizontal transfer of information that facilitates a rapid response to any external injury and ensures the community’s adaptation to a hostile environment (Papenfort and Bassler, 2016). Recent discoveries have identified signaling molecules that contribute to community quorum sensing, such as regulatory small RNAs (Papenfort and Vogel, 2010). Therefore, an antimicrobial agent endowed with RNase activity that can target bacterial quorum-sensing signaling might work as an antibiotic adjuvant. Indeed, the use of antibiotic adjuvants is one of the selected strategies to minimize the emergence and impact of resistance phenomena (Wright, 2016). Addition of adjuvants can lower the needed antibiotic dose to reach a therapeutic effect (Brown and Wright, 2016). Moreover, weakening the bacterial community cohesion would reduce the emergence and dissemination of resistant pathogenic strains (Starkey et al., 2014).

In this study, we have committed ourselves to evaluating the potential contribution of RNase catalytic activity in reducing the emergence of bacterial resistance. Toward this end, we have used an experimental evolution assay by exposing an *Acinetobacter baumannii* bacterial culture to increasing concentrations of colistin. Colistin (also called polymyxin E) is a non-ribosomal bacterial cyclic AMP only used in the clinics as a last resort to treat live-threatening infections, due to its reported toxicity. Here, we have tested the reduction of the antimicrobial resistance against colistin upon treatment with an engineered RNase construct that combines a high catalytic activity with specific antimicrobial properties.

## Materials and Methods

### Materials

The *A. baumannii* strain (CECT 452; ATCC 19606) and *Pseudomonas aeruginosa* strains (CECT 4122; ATCC 15692) are from the Spanish Type Culture Collection (CECT). The *Escherichia coli* BL21(DE3) strain and the pET11c plasmid are from Novagen. MRC-5 and HepG2 cells are from the American Type Cell Culture Collection (ATCCC). Mueller–Hinton broth, LPS and RNase A (Type XII) are from Sigma-Aldrich. 3-[4,5-dimethylthiazol-2-yl]-2,5-diphenyl tetrazolium bromide (MTT), Isopropyl β-D-1-thiogalactopyranoside (IPTG) and colistin are from Apollo Scientific. 1-aminonaphthalene-3,6,8-trisulfonate (ANTS), α,α′-dipyridinium p-xylene dibromide (DPX) and the fluorescent probe BODIPY TR cadaverine are from Molecular Probes. Toludine blue is from Merck. RNase 3/1 gene was purchased from NZYTech. 1,2-dioleoyl-*sn*-glycero-3-phosphocholine (DOPC) and 1,2-dioleoyl-*sn*-glycero-3-phosphoglycerol (DOPG) were from Avanti Polar Lipids. *E. coli* lipid extract was obtained as described (Folch et al., 1957). Human RNase 1 gene was a gift from Dr. Maria Vilanova, Universitat de Girona, Spain, and human RNase 3 sequence was taken from a previously synthesized gene (Boix et al., 1999).

### Protein Expression and Purification

The RNase 1, 3, and 3/1 genes were subcloned into the plasmid pET11c for prokaryote high yield expression in the *E. coli* BL21(DE3) strain. The recombinant protein was expressed and purified as previously described (Boix, 2001), with some modifications (Palmer and Wingfield, 2004). Briefly, bacteria were grown in terrific broth (TB), containing 400 μg/mL ampicillin. Recombinant protein was expressed after cell induction with 1 mM IPTG added when the culture showed an OD_600_ of 0.6. The cell pellet was collected after 4 h of culture at 37°C. Cells were resuspended in 10 mM Tris/HCl and 2 mM EDTA, pH 8 and 40 μg/mL of lysozyme, and sonicated after 30 min. The pellet was suspended in 25 mL of the same buffer with 1% triton X-100 and 1 M urea and was left stirring at room temperature for 30 min, before being centrifuged for 30 min at 22.000 × *g*. This procedure was repeated until the supernatant was completely transparent. In order to remove the triton X-100, 200 mL of 10 mM Tris-HCl pH 8.5, 2 mM EDTA was added to the pellet and centrifuged again for 30 min at 22.000 × *g*. The resulting pellet was suspended in 25 mL of Tris-acetate 100 mM, pH 8.5, 2 mM EDTA, 6 M guanidine hydrochloride, and 80 mM of DTT. The protein was then refolded for 72 h at 4°C by a rapid 100-fold dilution into 100 mM Tris/HCl, pH 8.5, 0.5 M of guanidinium chloride, and 0.5 M L-arginine, and oxidized glutathione (GSSG) was added to obtain a DTT/GSSG ratio of 4. The folded protein was then concentrated, buffer-exchanged against 150 mM sodium acetate, pH 5 and purified by cation-exchange chromatography on a Resource S (GE Healthcare) column equilibrated with the same buffer. The protein was eluted with a linear NaCl gradient from 0 to 2 M in 150 mM sodium acetate, pH 5. The protein purity was checked by SDS-PAGE and reverse-phase HPLC. Absence of unpaired Cys was confirmed by the Ellman’s reaction (Ellman, 1959).

### Circular Dichroism (CD)

Far-UV CD spectra were obtained from a Jasco-715 (Jasco), as previously described (Torrent et al., 2009a). The spectra were registered from 195 to 240 nm at room temperature. Data from four consecutive scans were averaged. Before reading, the sample was centrifuged at 10.000 × *g* for 5 min. Protein spectra were obtained at 6 μM in 5 mM sodium phosphate, pH 7.5, with a 0.2 cm path-length quartz cuvette. The percentage of secondary structure was estimated with Spectra Manager II, as described (Yang et al., 1986).

### Activity Staining Gel

Zymograms were performed following the method previously described (Bravo et al., 1994). 15% polyacrylamide-SDS gels were cast with 0.3 mg/mL of poly(C) (Sigma Aldrich). Then, 20 ng of RNase 1, 3, and 3/1 were loaded, and the gel was run at a constant current of 100 V for 1.5 h. Following, the SDS was removed from the gel with 10 mM Tris/HCl, pH 8, and 10% (v/v) isopropanol. The gel was then incubated during 1 h in the activity buffer (100 mM Tris/HCl, pH 8) to allow enzymatic digestion of the embedded substrate and then stained with 0.2% (w/v) toluidine blue in 10 mM Tris/HCl, pH 8, for 10 min. Positive bands appeared white against the blue background. The loading buffer had no 2-mercaptoethanol to facilitate recovery of active enzymes.

### Minimum Bactericidal Concentration (MBC) Determination

MBC_100_ was defined as the lowest protein/peptide concentration that completely eradicated bacterial cells. RNase 3/1 was serially diluted in HBS (HEPES 20 mm pH 7.4, NaCl 100 mM) in 96-well plate in a volume of 100 μL to achieve final concentrations from 20 to 0.02 μM. Then, 2 μL of an exponential phase subculture of *E. coli, A. baumannii* or *P. aeruginosa* was added, previously adjusted to give a final concentration of approximately 5 × 10^5^ colony-forming units (CFU)/mL in each well and the plate was incubated for 4 h at 37°C and 100 rpm. Finally, samples were plated onto LB (Condalab) Petri dishes and incubated at 37°C overnight. All the assays were performed in triplicate.

### Cytotoxicity Assay

Cytotoxicity was measured for the MRC-5 and HepG2 human cell lines using the MTT assay, as described previously (Pulido et al., 2018). Cells were grown in 5% CO_2_ at 37 °C with minimal essential medium supplemented with 10% fetal bovine serum (FBS). Cells were plated at 5 × 10^4^ cells/well in a 96-well plate and incubated overnight. Next, the medium was removed and serial dilutions of RNase 1, 3, and 3/1 were added at concentrations ranging from 200 to 0.2 μM in 100 μL of medium without serum. After 4 h of incubation, the medium was replaced with fresh medium containing 0.5 mg/mL MTT solution and the mixture was incubated for 2 h in 5% CO_2_ at 37 °C. The medium was then removed and formazan was dissolved by adding acidic isopropanol. The optical density (OD) was recorded by using a Victor^3^ plate reader (PerkinElmer, Waltham, MA) set at 550 and 630 nm as references. Reference absorbance at 630 nm was used to correct for nonspecific background values. Each experiment was repeated at least three times.

### LPS Binding Assay

The LPS-binding affinity was assessed using the fluorescent probe BODIPY TR cadaverine (BC) as described (Torrent et al., 2008). RNase 1, 3, and 3/1were serially diluted in a 96-well fluorescence plate from 20 to 0.02 μM in HEPES 10 mm pH 7.4. Then, LPS (10 μg/mL) and BC (10 μM) were added in the same buffer. Fluorescence measurements were performed on a Victor^3^ plate reader. The BC excitation wavelength was 580 nm, and the emission wavelength was 620 nm. Occupancy factor was calculated as described previously (Torrent et al., 2008).

### Liposome Preparation

Large unilamellar vesicles (LUVs) containing DOPC/DOPG (3:2 molar ratio, 1 mM stock concentration) or *E. coli* membrane lipids (5 mg/mL stock concentration) of a defined size were obtained from a vacuum drying lipid chloroform solution. After the chloroform evaporation, liposomes were suspended with 10 mM Tris/HCl, 20 mM NaCl, pH 7.4. The distribution and the mean hydrodynamic range of the liposomes in suspension was determined by dynamic light scattering (DLS) with a Zetasizer Nano ZS Malvern, and the data was analyzed by its built-in software (Zetasizer 7.02).

### Liposome Leakage

The ANTS/DPX liposome leakage fluorescence assay was performed as previously described (Pulido et al., 2016a). Briefly, a unique population of LUVs DOPC/DOPG (3:2) or *E. coli* liposomes was prepared to encapsulate a solution containing 12.5 mM ANTS, 45 mM DPX, 20 mM NaCl, and 10 mM Tris/HCl, pH 7.5 by applying three freeze-thaw cycles. The ANTS/DPX liposome stock suspension was diluted to 30 μM and incubated at 37°C for 1 h with RNase 1, 3, and 3/1, serially diluted from 20 to 0.015 μM in a microtiter fluorescence plate. Fluorescence measurements were performed on a Victor^3^ plate reader with an excitation wavelength of 386 nm and an emission wavelength of 535 nm. ED_50_ values were calculated by fitting the data to a dose–response curve with Origin 8.0.

### Synergy Determination Assay

The synergy test was carried out by determining the bacterial minimum inhibitory concentration (MIC) in Müller Hinton medium, as follows. 8 × 8 well plates were used to check the protein/colistin combinations. First, RNase 3/1 was serially diluted from 12 to 0.1 μM, in eppendorf tubes at a 10-fold the final concentration in 0.01% acetic acid (Wiegand et al., 2008). Then, colistin was serially diluted from 8 to 0.02 μM in Mueller–Hinton medium to a final volume of 90 μL, and 10 μL of the serially diluted protein aliquots were added in each corresponding well (except in the negative control, where only buffer was added). After that, 2 μL of a 1:3 diluted 24-h culture of *A. baumannii* were added to each well to achieve a final 1:150 bacterial dilution (approximately 2 × 10^5^ CFU/mL), and the plate was incubated overnight at 37°C and 100 rpm. Positive or negative bacterial growth was checked by optical density. All the assays were performed in triplicate.

### Antimicrobial Resistance Evolution Assay by Colistin Exposure

A fresh and isolated colony of *A. baumannii* was picked up and left to grow overnight in Mueller–Hinton broth. The next day, the 24-h culture (stationary phase) was diluted 1:150 times (approximately 2 × 10^7^ CFU/mL) and placed in a 96-well polypropylene plate. Before the inoculation of the bacteria, 90 μL of colistin in Mueller–Hinton medium were added to 80 wells in order to achieve the desired concentration in the final volume (100 μL). To 40 of these wells, 10 μL of 1 μM RNase 3/1 (to have a final concentration of 100 nM) in a cationic protein/peptide stabilizer solution (acetic acid at 0.01%) was added (Wiegand et al., 2008). To the other 40 wells, the same solution without protein was added. Finally, Mueller–Hinton medium with 10% of the stabilizer solution was added to the other 16 wells, as a negative control.

In the first experiment (Assay 1), the plate was placed overnight in an Infinite F Nano ^+^ (Tecan) plate reader at 37°C with orbital shaking, and the OD_600_ was recorded every 5 min. The day after, the cultures at the stationary phase were diluted three times and 2 μL of the diluted bacteria were inoculated to a new plate prepared as explained above, in order to have a 1:150 final dilution and incubated overnight to determine the bacterial growth profiles by DO_600nm_ monitoring. The first plate was stored at -80°C with 15% of glycerol for later determination of colistin MIC value. The dose of each day of colistin exposure was adjusted according to the observed bacterial growth; when no reduction of the bacterial growth was observed, the dose was increased; and, when bacteria growth reduction was observed, the dose was maintained.

In order to test if RNase 3/1 could also hinder the resistance acquisition of the bacteria in a colistin-pre-exposed strain, a second assay was performed, following a modification of a previously described assay (Lázár et al., 2018) (named as Assay 2). The plate preparation was performed as previously explained with the following modifications. Before starting the treatment protocol, a fresh isolated colony of *A. baumannii* was preexposed for three 24-h cycles to a 0.3 μM initial dose of colistin at the same conditions than the first exposure assay. Then, to each of 40 wells of the pre-exposed culture, the protein was added to a concentration of 1 μM, while the colistin dose was increased at 1.2 × serial incremental doses, keeping constant the protein concentration. The plates were incubated in an incubator at 37°C and 100 rpm. The OD_600_ was recorded after 24 h in a Victor^3^ plate reader.

### Minimum Inhibition Concentration (MIC) Determination

The MIC of each of the bacterial lineages generated during the resistance evolution assay was determined. MIC was defined as the lowest protein/peptide concentration that completely inhibited bacterial growth. Colistin was serially diluted from 64 to 0.5 μM in Mueller–Hinton. Next, 2 μL of a 1:3 diluted 24-h culture of each *A. baumannii* lineage were added to each well to achieve a final 1:150 bacterial dilution (approximately 2 × 10^7^ CFU/mL), and the plate was incubated overnight at 37°C and 100 rpm. Presence or absence of bacterial growth were checked by visual inspection.

### Statistical Analysis

Barlett’s test and Shapiro–Wilk’s method were used to study the variance homogeneity and normal distribution of the data, respectively. Due to unequal sample size among groups (unbalanced design), the Kruskall-Wallis non-parametric test followed by *post hoc* comparisons using Wilcoxon method were used for statistical comparison of continuous variable (time to reach exponential phase) for *A. baumannii* between and within the three different groups (culture bacteria treated with colistin, colistin + RNase 3/1 or non-treated control). Sample size (n) was 40 for the two treatments and 16 for the control group. Statistical significance was set at *p* < 0.05, and all statistical tests were 2-sided. Data are presented as means ± standard error. Statistical analysis and data visualization was performed using R Software 3.4.4 (R Core Team, 2018).

## Results

### RNase 3/1 Rational Design

**FIGURE 1:**
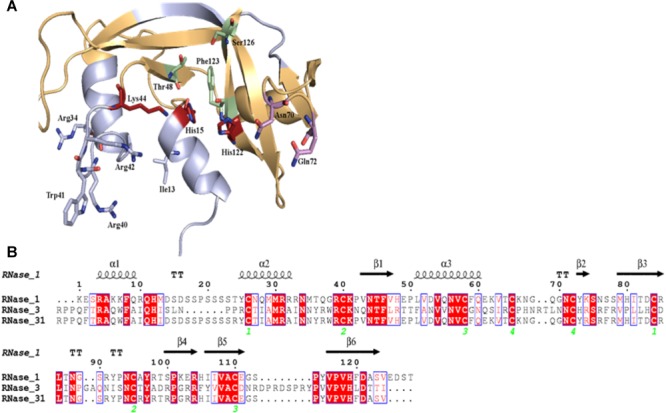
Design of RNase3/1 chimera. **(A)** Model of RNase 3/1 obtained by Modeler 9.12 (Webb and Sali, 2016). In green, B1 site. In pink, B2 site. In red, the active site. In beige, the RNase 1 skeleton. In light blue, the RNase 3 antimicrobial regions. **(B)** Alignment of RNase 1, 3 and 3/1 primary structures. The fully conserved amino acids are highlighted in red. The residues that are not conserved but have similar properties are marked with red letters. The secondary structure is indicated above the alignment. The green numbers indicate the disulphide bridge pairs. The alignment was done using *Clustal Omega* (Sievers and Higgins, 2018), and the image was obtained with *ESPript7* (http://espript.ibcp.fr/ESPript/) (Robert and Gouet, 2014).

RNase 3/1 has been designed in an effort to combine both the high bactericidal activity of human RNase 3 and the high catalytic activity of human RNase 1, according to previous structural-functional studies (Prats-Ejarque et al., unpublished results). Using the RNase 1 structure as a scaffold, antibacterial regions of RNase 3 were added. Specifically, we incorporated the N-terminal region of RNase 3, previously found to be related to antimicrobial activity (Torrent et al., 2009a, 2010, 2013; Sánchez et al., 2011), but preserved the Asp17-Ser26 flexible loop of RNase 1, considered essential for RNase 1 high catalytic activity (Doucet et al., 2009; Gagné et al., 2015). In addition, the cationic regions from Arg77 to Arg81 and Arg103 to Arg107, associated to RNase 3 bactericidal activity, were incorporated (Carreras et al., 2003). On the other hand, the residues that participate in the binding of the main and secondary bases of the RNA substrate, located at the 5′ and 3′ sides of the cleaved phosphodiester bond (B1 and B2, respectively), were kept according to the RNase 1 sequence. The resulting final sequence and the structure model are shown in Figure 1.

RNase 3/1 was successfully expressed in *E. coli* BL21(DE3) cells and purified from inclusion bodies at a final yield of 30 mg/L of bacterial culture. The proper 3D protein folding was checked by circular dichroism (Supplementary Figure S1), giving the characteristic secondary structure percentage distribution of the RNase A family (Fowler et al., 2011). Moreover, we confirmed by Ellman’s assay that all disulphide bonds were formed in the refolded protein and no free Cys residue was present (*A*_412 nm_ = 0.0003 and 0.001 for 4 and 8 μM of protein sample respect to control buffer).

**FIGURE 2:**
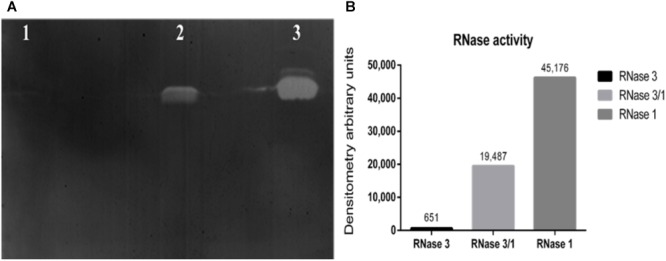
Catalytic characterization of RNase3/1 by zymogram. **(A)** Activity staining gel showing the degradation of poly(U) with 20 ng of each protein. RNase 3 (lane 1), RNase 3/1 (lane 2), and RNase 1 (lane 3). **(B)** Densitometric analysis of the bands of the activity staining gel. Quantification was done from a scan image using *Quantity One* software (Bio-Rad^®^).

Next, we assessed the protein catalytic activity using an activity staining gel. Results indicated that the RNase 3/1 hybrid displays a high catalytic efficiency toward a polynucleotide substrate. As shown in Figure 2, the catalytic activity of RNase 3/1 for polyuridine (poly(U)) is more than 30 times higher than that of RNase 3, almost reaching half of the catalytic activity of RNase 1.

In view of these results, we wanted to check whether the engineered structural changes that achieved an increase in catalytic activity in relation to RNase 3 had any detrimental effect on the hybrid proteins antimicrobial activity. Therefore, we determined the MBC for the hybrid and parental RNases. The results confirmed the acquisition of bactericidal activity of RNase 3/1 respect to the non-antimicrobial RNase 1; although a significant decrease of the hybrid bactericidal activity in comparison to RNase 3 is appreciated (Table 1).

**Table 1:** Bactericidal activity characterization of RNase3/1.

	MBC_100_ (μM)		
	*E. coli*	*A. baumannii*	*P. aeruginosa*
RNase 1	>20	>20	18.33 ± 2.89
RNase 3	1.88 ± 0.88	0.6 ± 0.07	0.6 ± 0.07
RNase 3/1	6.25 ± 2.17	6.25 ± 2.17	3.13 ± 1.08

*MBC_*100*_ was determined as the lowest concentration of peptide where no cells were detected by Colony Forming Units counting in HBS solution.*

Next, we evaluated whether our protein construct retained the high LPS-binding affinity observed for RNase 3 (Torrent et al., 2008; Pulido et al., 2012, 2016b). LPS-binding affinity was estimated by a displacement assay using the fluorescent BODIPY-cadaverine (BC) probe as previously described (Torrent et al., 2008). Results confirmed that RNase 3/1 retained a similar LPS-binding affinity to RNase 3 (Table 2 and Supplementary Figure S2).

**Table 2:** LPS-binding affinity and liposome leakage activity of RNases 1, 3 and 3/1.

	LPS binding (LBC_50_) (μM)	Liposome leakage (LC_20_) (μM)	
		DOPC:DOPG	*E. coli*
RNase 1	1.50 ± 0.28	N.D.	N.D.
RNase 3	0.38 ± 0.03	1.19 ± 0.19	1.87 ± 0.42
RNase 3/1	0.59 ± 0.02	1.18 ± 0.36	0.86 ± 0.18

*The LPS binding concentration at the 50% displacement (LBC_*50*_) values have been determined from a dose-response curve adjusted by *OriginPro 8* statistical software. Liposome leakage was measured as the protein concentration that induces 20% of liposome leakage (LC_*20*_). Experimental data plots are shown in Supplementary Figures S2, S3, respectively. N.D. indicates that no leakage was detected at the highest tested concentration (20 μM).*

In addition, the liposome leakage ability of the RNases was evaluated. As shown in Table 2, RNase 3/1 retains similar liposome leakage activity to RNase 3, while RNase 1 does not present any membrane disruption ability.

Finally, the potential cytotoxicity against host tissues was evaluated *in vitro* using two human cell lines: immortalized lung fibroblasts (MRC-5) and tumor hepatic cells (HepG2). The results indicated that RNase 3/1 shows no toxicity at the maximum concentration tested (200 μM) as observed for RNase 1 (Table 3).

**Table 3:** Cytotoxic activity characterization of RNase3/1.

	IC_50_ (μM)		
	RNase 1	RNase 3	RNase 3/1
Lung fibroblasts (MRC-5)	N.D.	>100	N.D.
Hepatocarcinoma epithelial cells (HepG2)	N.D.	134.66 ± 0.95	N.D.

*IC_*50*_ was determined as the concentration of protein where 50% of cells were still alive. Cytotoxicity was determined by the MTT assay. Each assay was performed in triplicate. N.D. means that, at the highest tested concentration (200 μM), no reduction of cell viability was detected.*

### RNase 3/1 Shows Synergy Activity With Colistin Against *A. baumannii*

**FIGURE 3:**
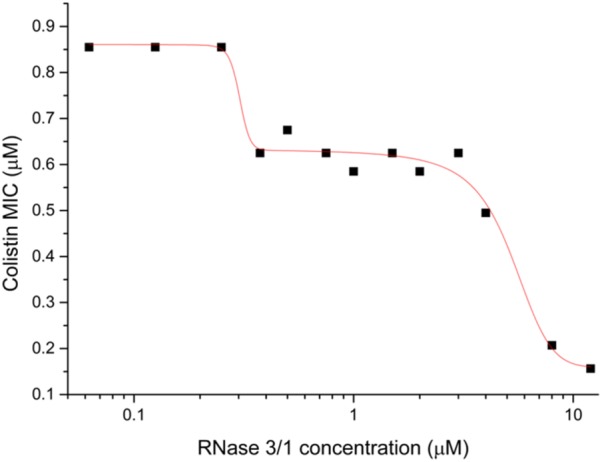
Biphasic dose-response fit of the variation of the MIC_100_ of colistin against *A. baumannii* as a function of the RNase 3/1 concentration. The non-linear fitting was done using *OriginLab 8*.

Next, we decided to test the potential synergistic activity of RNase 3/1 in combination with a common antimicrobial peptide used in the clinics, colistin. The analysis of the combinatorial effect of RNase 3/1 with colistin showed that the combination of colistin with RNase 3/1 improves significantly the MIC_100_ of colistin against *A. baumannii* (Figure 3). The MIC_100_ value for colistin alone in the assayed conditions was 0.9 ± 0.05 μM. The calculated RNase 3/1 concentration where the MIC for colistin is reduced by half was 1.35 ± 0.15 μM. Interestingly, we observe a biphasic dose-response profile, where the MIC for colistin reaches a first plateau in the presence of 0.3 to 5 μM of RNase 3/1 (∼1.5 × reduction of the original MIC value) and a second plateau at concentrations higher than 10 μM of RNase 3/1 (∼5 × reduction of the MIC value).

### Exposure of Bacterial Culture to RNase 3/1 Reduces the Acquisition of Bacterial Resistance to Colistin

**FIGURE 4:**
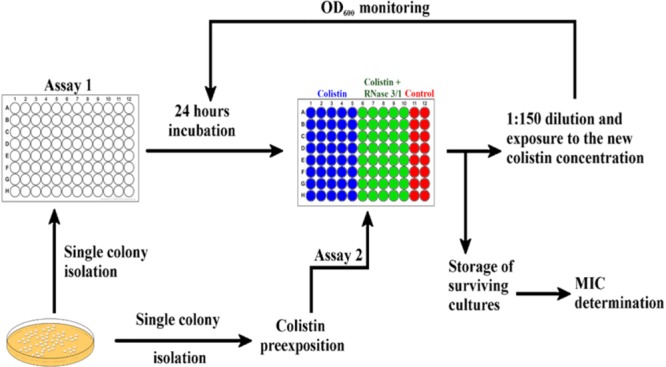
Graphical depiction of the experimental evolution of resistance assay protocol by culture exposure to colistin. 40 parallel lineages of *A. baumannii* were exposed to colistin or colistin + RNase 3/1. Assay 1 was started from a single colony without colistin preexposure incubated in the presence of 0.1 μM of RNase 3/1. Assay 2 was started from a culture pre-exposed to 0.3 μM of colistin during 3 days and addition of RNase 3/1 at 1 μM.

Positive synergy results encouraged us to test our hybrid RNase in an antimicrobial resistance evolution assay. Toward this end, we performed two different exposure protocols, as described in the methodology section, which we will refer to as Assays 1 and 2 (see Figure 4). In Assay 1, we exposed non-treated *A. baumannii* cells to either colistin alone or colistin supplemented with RNase 3/1 at 0.1 μM. RNase 3/1 final concentration was selected well below the minimum value required to display any potential synergy effect in combination with colistin (see Figure 3). The MIC value for RNase 3/1 in the assay conditions is ≥10 μM. During the experiment, in order to increase the selection pressure, we gradually increased the dose of colistin in accordance to the bacterial growth, while keeping the same initial RNase 3/1 concentration at 0.1 μM. The cultures of 40 parallel lineages were serially diluted and transferred for eleven consecutive cycles of 24-h each, corresponding to a total of about 330 generations as described (Moffatt et al., 2010). Colistin dose was adjusted according to the observed bacterial growth curves: the dose was increased when no effect on the bacterial growth was observed, and kept constant when a significant reduction of bacterial growth was registered. The growth of the bacteria following 1/150 dilution was monitored every 5 min and results were compared to parallel non-exposed control cultures.

**FIGURE 5:**
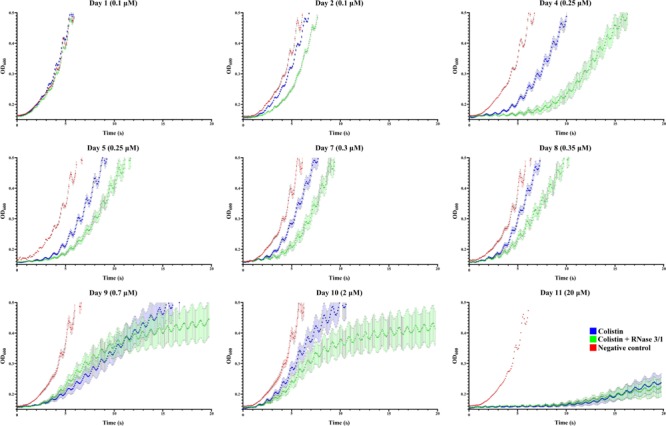
*A. baumannii* growth curves at representative days of exposure. Bacterial culture growth was monitored following 1/150 dilution by OD_600_ reading. Points represent the mean of all the lineages for each day and condition. In red is shown the negative control, in blue the colistin-exposed cultures and in green, the colistin + RNase 3/1 conditions. Error bars represent the standard error of the mean. A statistical analysis was applied to further evaluate the significance of the observed differences between the behavior of the cultures exposed only to colistin and the cultures exposed to both colistin and RNase 3/1. Taking as a reference the time needed for the bacteria to reach the exponential phase (OD_600_ = 0.4), we performed a comparison between cultures belonging to the same cycle (Figure 6B) and cultures of consecutive cycles within each condition (Supplementary Figures S4, S5).

Evaluation of the growth curves confirmed that the experimental evolution assay has been successful. The monitoring of culture optical density indicated that the exposure to increasing concentrations of colistin delayed bacterial growth (see Figure 5). Interestingly, the growth curve displacement is more pronounced by the addition of RNase 3/1.

**FIGURE 6:**
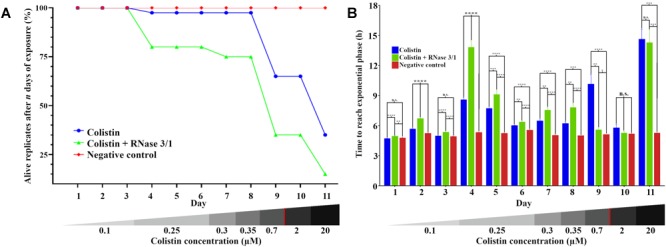
Evolution of the resistance acquisition of *A. baumannii* to colistin in the presence and absence of RNase 3/1. **(A)** Graphical representation of the surviving replicates after each day of exposure. **(B)** Graphical representation of the time needed for the surviving bacteria upon treatment to reach exponential phase (OD_600_ = 0.4). The remaining alive replicates are indicated following each day of exposure. Significance is calculated between the different conditions of the same day; n.s. means no significance between the compared samples, ^∗^*p* < 0.05, ^∗∗^*p* < 0.01, ^∗∗∗^*p* < 0.001, and ^∗∗∗∗^*p* < 0.0001 are indicated. The bottom red line indicates the calculated MIC of the colistin alone.

Moreover, addition of RNase 3/1 not only displaced the bacterial growth curve, producing a delay in the overall growth of the surviving bacterial lineages, but also resulted in increased mortality rate (see Table 4 and Figure 6A). Noteworthy, the cycles corresponding to the highest mortality rates (Day 9 and Day 11 in case of colistin alone; Day 4 and Day 11 in case of colistin combined with RNase 3/1) correlate with an increase in the time needed to reach the exponential phase. To note, at the 4th day of exposure, when the colistin dose was increased from 0.1 to 0.25 μM, we observed a marked decrease in growth in both colistin and colistin + RNase 3/1 conditions. In particular, the response to the increase of the colistin dose was most prominent in the RNase 3/1 supplemented condition, achieving, at 0.25 μM (Day 4), a 20% of mortality versus only a 2.5% in the cultures exposed only to colistin (Figure 6A). Likewise, we observed a pronounced shift of the growth curves (Figure 5, 6B).

**Table 4:** Evolution of the resistance acquisition of *A. baumannii* to colistin, considering the number of replicates alive after each day of exposure.

	Alive replicates after *n* days of exposure (%)										
	D1	D2	D3	D4	D5	D6	D7	D8	D9	D10	D11
Colistin	100	100	100	97.5	97.5	97.5	97.5	97.5	65	65	35
Colistin											
+ RNase 3/1	100	100	100	80	80	80	75	75	35	35	15
Negative control	100	100	100	100	100	100	100	100	100	100	100
Colistin dose	0.1 μM			0.25 μM			0.3 μM	0.35 μM	0.7 μM	2 μM	20 μM

*The initial number of replicate lineages was 40. Colistin dose was gradually increased according to bacterial growth, as detailed in the methodology. The colistin dose used in each exposure is indicated in the bottom row. RNase 3/1, when used, was kept constant at 0.1 μM.*

A second peak of mortality was visualized between days 8 and 9 (corresponding to 0.35 μM to 0.7 μM of colistin dose increase). At this point, only 35% of the original strains exposed to colistin + RNase 3/1 survived, versus a 65% of survival for colistin alone. Interestingly, although the condition exposed to colistin alone experimented a significant delay of growth (Figure 6B), this was not the case for the RNase 3/1 supplemented condition, suggesting that the surviving strains had already acquired resistance to that dose of colistin.

Eventually, a shock dose of colistin (10 × the dose of the previous day) was applied at day 11, triggering a high mortality and a strong delay of the growth of the surviving strains. Following this shock dose, the surviving rate of the lineages exposed to colistin and RNase 3/1 was less than half the observed in the cultures exposed to colistin alone (35–15% of survival, respectively), as shown in Figure 6A and Table 4.

**FIGURE 7:**
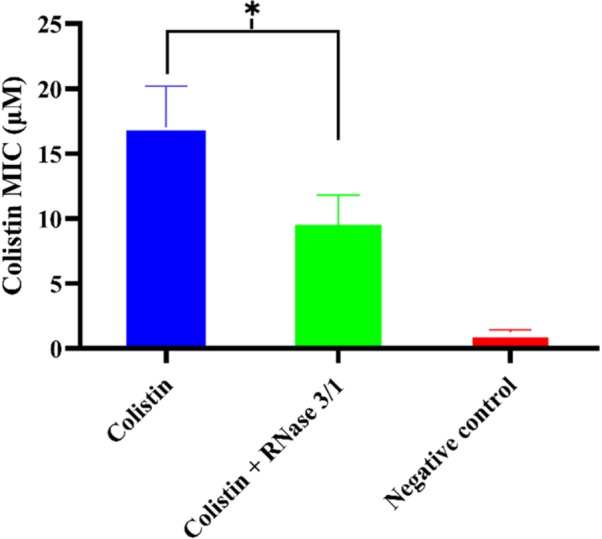
Comparison of the calculated average mean MIC value following Assay 1 treatment for RNase 3/1-supplemented versus non-supplemented bacterial strains exposed to colistin. Error bars show the standard error of the mean. ^∗^indicates that the *p*-value is < 0.05.

On the other hand, results highlighted an overall reduction by half in the calculated average MIC mean value for colistin in the bacterial cultures supplemented with RNase 3/1 (Figure 7).

**FIGURE 8:**
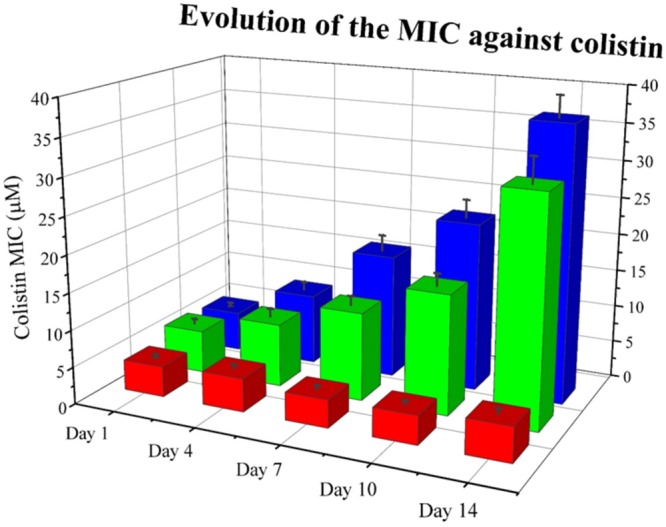
Evolution of the mean MIC value for colistin of each assayed condition in Assay 2. Overall reduction of calculated MIC for colistin is observed for samples supplemented with RNase 3/1. Red corresponds to the negative control, blue to the colistin alone condition and green to the colistin + RNase 3/1 condition. Data from selected days is shown. The raw data used to produce the figure is shown in Supplementary Figure S6. Error bars show the standard error of the mean.

Next, we wanted to explore whether RNase 3/1 addition to colistin could also hinder the bacterial resistance acquisition to a strain that had previously acquired resistance to colistin. With this goal in mind, we incubated an *A. baumannii* culture for 3 days in the presence of 0.3 μM of colistin, and then, we performed a second experimental evolution assay using this strain, with acquired resistance to colistin, as a starting seed (see Assay 2 in Figure 4). Then, the cultures were exposed to either colistin alone or colistin supplemented with RNase 3/1 at 1 μM final protein concentration. In this experiment, RNase 3/1 concentration was added at 1 μM to ensure a positive response when tested in combination with colistin (see Figure 3). The 40 parallel lineages were exposed to gradually increasing concentrations of colistin (1.2-fold following each transfer cycle of 24 h, starting from an initial concentration of 0.3 μM, see corresponding daily concentrations in Supplementary Figure S6). The tested colistin range (from 0.3 to 3.21 μM) ensured the induction of bacterial resistance and avoided massive cell mortality. Results indicated that the average increase of the MIC for colistin following 14 cycles of 24 h (about 420 generations) was significantly decreased for the condition supplemented with RNase 3/1 in comparison to the non-supplemented sample (Figure 8 and Supplementary Figure S6).

## Discussion

The design of novel strategies for the eradication of bacterial resistance forms is one of the main concerns of the global health organization and is among the top priority lines of action of the current research policy. The extensive use and abuse of conventional antibiotics during the last decades have generated a population of resistant bacteria. This phenomenon has been aggravated by the fact that most commonly used antibiotics nowadays are based on bacterial secreted natural products. Indeed, the pharmaceutical industry has deeply exploited the naturally produced self-defense toxins that bacteria secrete to fight against competitor strains. Following billions of years of co-evolution, a reservoir of resistance genes has been created that can confer bacterial protection to most conventional antibiotics. In addition, we cannot underestimate the inherent ability of bacterial cells to generate diversity. This process can constantly enrich the bacterial resistome pool, which is easily mobilized in case of emergency (Blair et al., 2015; Knoppel et al., 2017). Last but not least, cooperative mechanisms are taking place between the individual cells of the bacterial community and can provide a collective antimicrobial resistance profile (Vega and Gore, 2014). The intercellular network organization can play a prominent role under stressful conditions and can facilitate an appropriate response to external injuries. Therefore, the blockage of bacterial signaling systems should significantly reduce the emergence of resistance mechanisms. Noteworthy, the horizontal exchange of material between the integrands of the community is mainly delivered by bacterial extracellular vesicles. Bacterial extracellular vesicles ensure the exchange of proteins and nucleic acids (Kulp and Kuehn, 2010; Rumbo et al., 2011; Schwechheimer and Kuehn, 2015; Jan, 2017) and mediate the rapid adaption of the community to hostile environments. Interestingly, the transfer of RNA molecules has been identified (Guerrero-Mandujano et al., 2017) to participate in transcription regulation mechanisms (Sjostrom et al., 2015; Koeppen et al., 2016). Overall, the search of novel drugs that target the bacterial community signaling mechanisms, also referred as quorum sensing inhibitors, is gaining prominence in the pharmaceutical research field.

Within this context, a compound endowed with RNase activity would be able to interfere in the horizontal exchange of RNA and thereby attack the community cohesion. To note, the use of the enzymatic activities of bacteriocin toxins was proposed as a strategy to weaken the biofilm community viability (Mathur et al., 2018). Bacteriocins are antimicrobial proteins secreted by bacteria that specifically target quorum sensing systems. Among these proteins we can find enzymes with protease, DNase and RNase activities that facilitate biofilm removal. Interestingly, recent literature reveals the structure fold homology between vertebrate secretory RNases and bacterial RNases that work as inter-strain competition toxins (Batot et al., 2017; Cuthbert et al., 2018). A parallelism can be established between bacterial RNases secreted as virulence toxins and secretory RNases expressed by innate cells in vertebrates (Lu et al., 2018). An interesting hypothesis puts forward the scenario where the proteins secreted by granulocytes could provide a link between a simple local response and a systemic organized signaling process associated to the increase in size and complexity of multicellular organisms. In this context, the host innate cells would have retained similar self-defense mechanisms as displayed by unicellular organisms. The ancestral role of bacterial virulence toxins would have derived to a more specialized role toward body fluid protection against infection (Lee and Lee, 2005). Understanding the similarities between unicellular self-defense and mammalian innate cell mechanisms can provide novel strategies to take profit of our immune response system. Undoubtedly, our own host defense system constitutes one of the most attractive sources of novel antimicrobial agents.

In this work, we have explored the potentiality of an engineered RNase construct derived from a human antimicrobial member of the vertebrate-specific RNase A superfamily. Based on our previous structural-functional studies we have engineered a hybrid RNase that combines a high catalytic activity together with specific antimicrobial properties. The RNase 3/1 form conserves the RNase 1 scaffold that provides the essential requirements for an elevated catalytic activity (Nogués et al., 1998; Raines, 1998; Doucet et al., 2009; Gagné et al., 2015) and encompasses the key features of RNase 3 antimicrobial activity (Figure 1) (Carreras et al., 2003; Boix et al., 2008, 2012; Torrent et al., 2009a, 2013; Pulido et al., 2013). Experimental results confirmed that our new construct successfully combines a high catalytic activity (Figure 2) together with a high bactericidal activity for all tested Gram-negative species (Table 1). In addition, the RNase 3/1 variant retained the characteristic high affinity for LPS of RNase 3 (Torrent et al., 2008) together with its membrane leakage activity (Torrent et al., 2007, 2009b) (Table 2). On the other hand, RNase 3/1 shares with RNase 1 its non-toxicity to the tested human cell lines (Table 3). Indeed, human secretory RNases’ harmlessness is ensured thanks to the presence in the cytosol of all human tissue cells of the RNase inhibitor (RI), a horseshoe-shaped protein that binds to the human secretory RNase members (Lomax et al., 2014) in a 1:1 stoichiometry with an unusually high affinity (at the fM-nM range). The RI is ubiquitous and protects the host cells from the potential toxicity of secretory RNases (Dickson et al., 2005). Therefore, our RNase chimera combines a high affinity for bacterial membranes together with an elevated catalytic efficiency and no toxicity to the host.

Here, we have investigated the potency of our lead construct to work as an adjuvant molecule and reduce the emergence of bacterial resistance to colistin, an antimicrobial peptide, commonly used in the clinics. Colistin targets bacterial cells by a direct action at the cell membrane and bacterial wall, and is mostly used to treat difficult infections, such as the ones caused by Gram-negative multidrug resistant strains (Stein and Raoult, 2002; Michalopoulos et al., 2005; Sabuda et al., 2008; Mendelson et al., 2018). However, colistin can accumulate in many body tissues, such as kidney and liver, and shows considerable collateral toxicity to the host in the long term, so it is only prescribed as a last resort in critically ill patients (Stein and Raoult, 2002; Michalopoulos et al., 2005; Sabuda et al., 2008; Mendelson et al., 2018).

The use of drug combinations is currently attracting attention in the fight against antimicrobial resistance (Tabbene et al., 2015; Brochado et al., 2018; Lázár et al., 2018). The co-administration of an adjuvant molecule can significantly lower the required effective dose to treat an infection. In the same vein, our present data using *A. baumannii* cultures indicate that by combining the engineered RNase construct with colistin we can reduce its effective dose (Figure 3). Interestingly, this effect shows a biphasic dose-response profile, with the colistin MIC reaching a first plateau when it is supplemented with 0.3–5 μM of RNase 3/1 (∼1.5 × decrease of the MIC value) and a second plateau at concentrations of RNase 3/1 higher than 10 μM (∼5 × decrease of the MIC). We can hypothesize that this two-step reduction of the MIC for colistin is due to a dual mechanism of action of our construct, where a first event is dependent on the protein enzymatic activity and a second process is triggered by a direct action against the bacterial cell wall.

Encouraged by the results of the synergy assay we decided to analyze whether the RNase addition could target the emergence of bacterial resistance. Toward this end, we set up an *in vitro* evolution assay where resistance against colistin was generated following the culture exposure to increasing concentrations of the antimicrobial peptide. Forty parallel lineages of *A. baumannii* cultures were exposed to increasing concentrations of colistin from 0.1 to 20 μM. After 330 generations of exposure, we observed a 1.8-fold reduction of the calculated MIC value for colistin by addition of RNase 3/1. Moreover, when the experimental evolution assay was started using a colistin-resistant strain, the acquisition of further resistance to colistin was also significantly delayed by supplementation of the RNase 3/1 protein.

Results highlight that the RNase 3/1 chimera can significantly reduce the emergence of bacterial resistance. The construct’s efficacy to inhibit the emergence of *A. baumannii* resistance to colistin at a concentration below its effective bactericidal activity indicates that the mechanism of action of RNase 3/1 is probably due to a combination of the protein specific properties and not to a direct bactericidal action. The present experimental results suggest that the RNase may act as a double-edged sword, with both enzymatic activity and membrane disruption/LPS binding being responsible for the observed phenomenon. Further work is currently ongoing to determine the specific mechanism underlying RNase 3/1 reduction of the emergence of bacterial resistance and ideate additional strategies to enhance its potential adjuvant properties.

Although further studies will be necessary to confirm this hypothesis, we speculate that the RNase activity might interfere with the bacterial community quorum sensing. Since bacterial resistance to cell-wall targeting antibiotics is often associated to alterations of the outer membrane composition (Moffatt et al., 2010; Lázár et al., 2018), an antimicrobial agent able to block bacterial intercellular communication should reduce the cell survival adaptation stratagems. In particular, our RNase 3/1 chimera, with a high binding affinity to LPS, a positive liposome leakage and high enzymatic activity, might block the inter-bacterial communication. However, further testing with other RNase constructs are mandatory to understand the whole process. Likewise, we are planning to analyze the potential combinatorial effect of our RNase construct in the presence of other families of conventional antibiotics, to discard any specific synergistic effect unique to the antimicrobial mechanism of colistin. In addition, to fully interpret our results, it is important to analyze whether the antibiotic resistome profile differs between the control exposed to colistin and the RNase 3/1 treated bacterial cultures.

Our results emphasize once more the applicability of host defense proteins to design novel anti-infective therapies. One of the main sources of effective adjuvants is the host defense arsenal of proteins and peptides. We find several examples of host defense peptides that are not antimicrobial by themselves but can boost the host’s innate immunity. Some of them are currently on clinical trials for the development of novel adjuvants (Hancock et al., 2012). Bearing in mind that antibiotic resistance mechanisms might be palliated but are probably impossible to fully eradicate (Wright, 2016), alternative strategies to conventional therapies are urgently needed. Drug combination is nowadays one of the best choices to combat multidrug resistance (Brochado et al., 2018). Antibiotic adjuvants can explore synergy events and significantly reduce the effective antibiotic dose (Wright, 2016). Although it would be necessary to fully characterize the mechanism through which RNase 3/1 hinders the bacterial resistance acquisition, RNases and their derivatives appear as new lead compounds in the fight against antimicrobial resistance.

## Conclusion

Targeting RNA to fight bacterial resistance is a promising approach toward the development of alternative antibiotic and/or adjuvant drugs. Our results highlight the ability of an RNase hybrid protein (RNase 3/1) that combines a high catalytic activity with specific antimicrobial properties, to display synergic activity when combined with colistin. Colistin is an antimicrobial cyclic peptide that is frequently used as a last resort antibiotic to treat multidrug resistant bacteria in severe infections but has some toxic side effects. The addition of our RNase lead compound can significantly reduce the effective antimicrobial dose of colistin. Moreover, we have demonstrated that the RNase 3/1 construct can efficiently target the bacterial antimicrobial resistance evolved upon colistin exposure. After 330 generations of exposure to colistin, we observed about a twofold reduction of the calculated MIC value when supplementing the culture with the RNase 3/1 construct. The present data anticipate the potential development of RNase-based lead molecules as antibiotic adjuvant candidates.

## Author Contributions

EB and GP-E conceived and designed the experiments. FA-I, GP-E, HL, and JL performed the experimental work. EB, FA-I, GP-E, and JL analyzed the data. EB and GP-E drafted the manuscript. EB, FA-I, GP-E, and JL revised the final manuscript. All authors approved the final version of the manuscript.

## Conflict of Interest Statement

The authors declare that the research was conducted in the absence of any commercial or financial relationships that could be construed as a potential conflict of interest.

## References

[B1] Arranz-Trullén J. Lu L. Pulido D. Bhakta S. Boix E. (2017). Host antimicrobial peptides: the promise of new treatment strategies against tuberculosis. *Front. Immunol.* 8:1499 10.3389/fimmu.2017.01499 PMC5681943 29163551

[B2] Bahar A. A. Ren D. (2013). Antimicrobial peptides. *Pharmaceuticals* 6 1543–1575. 10.3390/ph6121543 24287494 PMC3873676

[B3] Batot G. Michalska K. Ekberg G. Irimpan E. M. Joachimiak G. Jedrzejczak R. (2017). The CDI toxin of yersinia kristensenii is a novel bacterial member of the Rnase A superfamily. *Nucleic Acids Res.* 45 5013–5025. 10.1093/nar/gkx230 28398546 PMC5435912

[B4] Blair J. M. A. Webber M. A. Baylay A. J. Ogbolu D. O. Piddock L. J. V. (2015). Molecular mechanisms of antibiotic resistance. *Nat. Rev. Microbiol.* 13 42–51. 10.1038/nrmicro3380 25435309

[B5] Boix E. (2001). “Eosinophil cationic protein,” in *Methods in Enzymology* ed. Colowick S. P. (Amsterdam: Elsevier) 287–305. 10.1016/s0076-6879(01)41159-1 11582785

[B6] Boix E. Nikolovski Z. Moiseyev G. P. Rosenberg H. F. Cuchillo C. M. Nogués M. V. (1999). Kinetic and product distribution analysis of human eosinophil cationic protein indicates a subsite arrangement that favors exonuclease-type activity. *J. Biol. Chem.* 274 15605–15614. 10.1074/jbc.274.22.15605 10336457

[B7] Boix E. Nogués M. V. (2007). Mammalian antimicrobial proteins and peptides: overview on the RNase A superfamily members involved in innate host defence. *Mol. Biosyst.* 3 317–335. 10.1039/b617527a 17460791

[B8] Boix E. Salazar V. A. Torrent M. Pulido D. Nogués M. V. Moussaoui M. (2012). Structural determinants of the eosinophil cationic protein antimicrobial activity. *Biol. Chem.* 393 801–815. 10.1515/hsz-2012-0160 22944682

[B9] Boix E. Torrent M. Sánchez D. Nogués M. V. (2008). The antipathogen activities of eosinophil cationic protein. *Curr. Pharm. Biotechnol.* 9 141–152. 10.2174/138920108784567353 18673279

[B10] Brandenburg L. O. Merres J. Albrecht L. J. Varoga D. Pufe T. (2012). Antimicrobial peptides: multifunctional drugs for different applications. *Polymers* 4 539–560. 10.3390/polym4010539

[B11] Bravo J. Fernandez E. Ribo M. Dellorens R. Cuchillo C. M. (1994). A versatile negative-staining ribonuclease zymogram. *Anal. Biochem.* 219 82–86. 10.1006/abio.1994.1234 7520217

[B12] Brochado A. R. Telzerow A. Bobonis J. Banzhaf M. Mateus A. Selkrig J. (2018). Species-specific activity of antibacterial drug combinations. *Nature* 559 259–263. 10.1038/s41586-018-0278-9 29973719 PMC6219701

[B13] Brogden K. A. (2005). Antimicrobial peptides: pore formers or metabolic inhibitors in bacteria? *Nat. Rev. Microbiol.* 3 238–250. 10.1038/nrmicro1098 15703760

[B14] Brown E. D. Wright G. D. (2016). Antibacterial drug discovery in the resistance era. *Nature* 529 336–343. 10.1038/nature17042 26791724

[B15] Carreras E. Boix E. Rosenberg H. F. Cuchillo C. M. Nogués M. V. (2003). Both aromatic and cationic residues contribute to the membrane-lytic and bactericidal activity of eosinophil cationic protein. *Biochemistry* 42 6636–6644. 10.1021/bi0273011 12779318

[B16] Casciaro B. Loffredo M. R. Luca V. Verrusio W. Cacciafesta M. Mangoni M. L. (2018). Esculentin-1a derived antipseudomonal peptides: limited induction of resistance and synergy with aztreonam. *Protein Pept. Lett.* 25 1–8. 10.2174/0929866525666181101104649 30381056

[B17] Cuthbert B. J. Burley K. H. Goulding C. W. (2018). introducing the new bacterial branch of the rnase a superfamily. *RNA Biol.* 15 9–12. 10.1080/15476286.2017.1387710 29099294 PMC5786019

[B18] Dickson K. A. Haigis M. C. Raines R. T. (2005). Ribonuclease inhibitor: structure and function. *Prog. Nucleic Acid Res. Mol. Biol.* 80 349–374. 16164979 10.1016/S0079-6603(05)80009-1 PMC2811166

[B19] Doucet N. Watt E. D. Loria J. P. (2009). The flexibility of a distant loop modulates active site motion and product release in ribonuclease A. *Biochemistry* 48 7160–7168. 10.1021/bi900830g 19588901 PMC2741010

[B20] Ellman G. L. (1959). Tissue sulfhydryl groups. *Arch. Biochem. Biophys.* 82 70–77. 10.1016/0003-9861(59)90090-6 13650640

[B21] Folch J. Lees M. Sloane Stanley G. H. (1957). A simple method for the isolation and purification of total lipides from animal tissues. *J. Biol. Chem.* 226 497–509. 10.1016/j.ultrasmedbio.2011.03.005 13428781

[B22] Fowler C. B. Evers D. L. O’Leary T. J. Mason J. T. (2011). Antigen retrieval causes protein unfolding: evidence for a linear epitope model of recovered immunoreactivity. *J. Histochem. Cytochem.* 59 366–381. 10.1369/0022155411400866 21411808 PMC3201144

[B23] Gagné D. French R. L. Narayanan C. Simonović M. Agarwal P. K. Doucet N. (2015). Perturbation of the conformational dynamics of an active-site loop alters enzyme activity. *Structure* 23 2256–2266. 10.1016/j.str.2015.10.011 26655472 PMC4680847

[B24] Guerrero-Mandujano A. Hernández-Cortez C. Ibarra J. A. Castro-Escarpulli G. (2017). The outer membrane vesicles: secretion system type zero. *Traffic* 18 425–432. 10.1111/tra.12488 28421662

[B25] Gupta S. K. Haigh B. J. Griffin F. J. Wheeler T. T. (2012). The mammalian secreted RNases: mechanisms of action in host defence. *Innate Immun.* 19 86–97. 10.1177/1753425912446955 22627784

[B26] Hancock R. E. W. Diamond G. (2000). The role of cationic antimicrobial peptides in innate host defences. *Trends Microbiol.* 8 402–410. 10.1016/s0966-842x(00)01823-0 10989307

[B27] Hancock R. E. W. Nijnik A. Philpott D. J. (2012). Modulating immunity as a therapy for bacterial infections. *Nat. Rev. Microbiol.* 10 243–254. 10.1038/nrmicro2745 22421877

[B28] Hancock R. E. W. Sahl H. G. (2006). Antimicrobial and host-defense peptides as new anti-infective therapeutic strategies. *Nat. Biotechnol.* 24 1551–1557. 10.1038/nbt1267 17160061

[B29] Jan A. T. (2017). Outer membrane vesicles (OMVs) of gram-negative bacteria: a perspective update. *Front. Microbiol.* 8:1053 10.3389/fmicb.2017.01053 PMC5465292 28649237

[B30] Knoppel A. Nasvall J. Andersson D. I. (2017). Evolution of antibiotic resistance without antibiotic exposure. *Antimicrob. Agents Chemother.* 61 1–5. 10.1128/AAC.01495-17 PMC5655081 28893783

[B31] Koczera P. Martin L. Marx G. Schuerholz T. (2016). The ribonuclease a superfamily in humans: canonical RNases as the buttress of innate immunity. *Int. J. Mol. Sci.* 17:E1278 10.3390/ijms17081278 PMC5000675 27527162

[B32] Koeppen K. Hampton T. H. Jarek M. Scharfe M. Gerber S. A. Mielcarz D. W. (2016). A novel mechanism of host-pathogen interaction through sRNA in bacterial outer membrane vesicles. *PLoS Pathog.* 12:e100567 10.1371/journal.ppat.1005672 PMC4905634 27295279

[B33] Kulp A. Kuehn M. J. (2010). Biological functions and biogenesis of secreted bacterial outer membrane vesicles. *Annu. Rev. Microbiol.* 64 163–184. 10.1146/annurev.micro.091208.073413 20825345 PMC3525469

[B34] Lai Y. Gallo R. L. (2009). AMPed up immunity: how antimicrobial peptides have multiple roles in immune defense. *Trends Immunol.* 30 131–141. 10.1016/j.it.2008.12.003 19217824 PMC2765035

[B35] Lázár V. Martins A. Spohn R. Daruka L. Grézal G. Fekete G. (2018). Antibiotic-resistant bacteria show widespread collateral sensitivity to antimicrobial peptides. *Nat. Microbiol.* 3 718–731. 10.1038/s41564-018-0164-0 29795541 PMC6544545

[B36] Lee J. J. Lee N. A. (2005). Eosinophil degranulation: an evolutionary vestige or a universally destructive effector function? *Clin. Exp. Allergy* 35 986–994. 10.1111/j.1365-2222.2005.02302.x 16120079

[B37] Lohrasbi V. Talebi M. Bialvaei A. Z. Fattorini L. Drancourt M. Heidary M. (2018). Trends in the discovery of new drugs for *Mycobacterium tuberculosis* therapy with a glance at resistance. *Tuberculosis* 109 17–27. 10.1016/j.tube.2017.12.002 29559117

[B38] Lomax J. E. Bianchetti C. M. Chang A. Phillips G. N. Fox B. G. Raines R. T. (2014). Functional evolution of ribonuclease inhibitor: insights from birds and reptiles. *J. Mol. Biol.* 426 3041–3056. 10.1016/j.jmb.2014.06.007 24941155 PMC4219644

[B39] Lu L. Li J. Moussaoui M. Boix E. (2018). Immune modulation by human secreted RNases at the extracellular space. *Front. Immunol.* 9:1012 10.3389/fimmu.2018.01012 PMC5964141 29867984

[B40] Mathur H. Field D. Rea M. C. Cotter P. D. Hill C. Ross R. P. (2018). Fighting biofilms with lantibiotics and other groups of bacteriocins. *NPJ Biofilms Microbiomes* 4:9 10.1038/s41522-018-0053-6 PMC5908865 29707229

[B41] Mendelson M. Brink A. Gouws J. Mbelle N. Naidoo V. Pople T. (2018). The One Health stewardship of colistin as an antibiotic of last resort for human health in South Africa. *Lancet Infect. Dis.* 18 e288–e294. 10.1016/S1473-3099(18)30119-1 29673734

[B42] Michalopoulos A. S. Tsiodras S. Rellos K. Mentzelopoulos S. Falagas M. E. (2005). Colistin treatment in patients with ICU-acquired infections caused by multiresistant Gram-negative bacteria: the renaissance of an old antibiotic. *Clin. Microbiol. Infect.* 11 115–121. 10.1111/j.1469-0691.2004.01043.x 15679485

[B43] Moffatt J. H. Harper M. Harrison P. Hale J. D. F. Vinogradov E. Seemann T. (2010). Colistin resistance in *Acinetobacter baumannii* is mediated by complete loss of lipopolysaccharide production. *Antimicrob. Agents Chemother.* 54 4971–4977. 10.1128/AAC.00834-10 20855724 PMC2981238

[B44] Mustazzolu A. Borroni E. Cirillo D. M. Giannoni F. Iacobino A. Fattorini L. (2018). Trend in rifampicin-, multidrug- and extensively drug-resistant tuberculosis in Italy, 2009–2016. *Eur. Respir. J.* 52:1800070 10.1183/13993003.00070-2018 29724919

[B45] Nakatsuji T. Gallo R. L. (2012). Antimicrobial peptides: old molecules with new ideas. *J. Invest. Dermatol.* 132 887–895. 10.1038/jid.2011.387 22158560 PMC3279605

[B46] Nogués M. V. Moussaoui M. Boix E. Vilanova M. Ribó M. Cuchillo C. M. (1998). The contribution of noncatalytic phosphate-binding subsites to the mechanism of bovine pancreatic ribonuclease A. *Cell. Mol. Life Sci.* 54 766–774. 10.1007/s000180050205 9760985 PMC11147180

[B47] Palmer I. Wingfield P. T. (2004). “Preparation and extraction of insoluble (inclusion-body) proteins from *Escherichia coli*,” in *Current Protocols in Protein Science* ed. Coligan J.E. (Hoboken, NJ: John Wiley & Sons, Inc.). 10.1002/0471140864.ps0603s38 PMC3518028 18429271

[B48] Papenfort K. Bassler B. L. (2016). Quorum sensing signal-response systems in gram-negative bacteria. *Nat. Rev. Microbiol.* 14 576–588. 10.1038/nrmicro.2016.89 27510864 PMC5056591

[B49] Papenfort K. Vogel J. (2010). Regulatory RNA in bacterial pathogens. *Cell Host Microbe* 8 116–127. 10.1016/j.chom.2010.06.008 20638647

[B50] Perron G. G. Zasloff M. Bell G. (2006). Experimental evolution of resistance to an antimicrobial peptide. *Proc. R. Soc. London B* 273 251–256. 10.1098/rspb.2005.3301 PMC1560030 16555795

[B51] Pulido D. Arranz-Trullén J. Prats-Ejarque G. Velázquez D. Torrent M. Moussaoui M. (2016a). Insights into the antimicrobial mechanism of action of human rnase6: structural determinants for bacterial cell agglutination and membrane permeation. *Int. J. Mol. Sci.* 17:552 10.3390/ijms17040552 PMC4849008 27089320

[B52] Pulido D. Garcia-Mayoral M. F. Moussaoui M. Velázquez D. Torrent M. Bruix M. (2016b). Structural basis for endotoxin neutralization by the eosinophil cationic protein. *FEBS J.* 283 4176–4191. 10.1111/febs.13915 27696685

[B53] Pulido D. Moussaoui M. Andreu D. Nogués M. V. Torrent M. Boix E. (2012). Antimicrobial action and cell agglutination by the eosinophil cationic protein are modulated by the cell wall lipopolysaccharide structure. *Antimicrob. Agents Chemother.* 56 2378–2385. 10.1128/AAC.06107-11 22330910 PMC3346588

[B54] Pulido D. Torrent M. Andreu D. Nogués M. V. Boix E. (2013). Two human host defense ribonucleases against mycobacteria, the eosinophil cationic protein (RNase 3) and RNase 7. *Antimicrob. Agents Chemother.* 57 3797–3805. 10.1128/AAC.00428-13 23716047 PMC3719706

[B55] Pulido D. Villalba C. Prats-Ejarque G. Albacar M. Moussaoui M. Andreu D. (2018). Positional scanning library applied to the human eosinophil cationic protein/RNase 3 N-terminus reveals novel and potent antibiofilm peptides. *Eur. J. Med. Chem.* 152 590–599. 10.1016/j.ejmech.2018.05.012 29763807

[B56] R Core Team (2018). *R: A Language and Environment for Statistical Computing*. Vienna: R Foundation for Statistical Computing.

[B57] Raines R. T. (1998). Ribonuclease A. *Chem. Rev.* 98 1045–1066. 10.1021/cr960427h 11848924

[B58] Robert X. Gouet P. (2014). Deciphering key features in protein structures with the new ENDscript server. *Nucleic Acids Res.* 42 320–324. 10.1093/nar/gku316 PMC4086106 24753421

[B59] Rumbo C. Fernández-Moreira E. Merino M. Poza M. Mendez J. A. Soares N. C. (2011). Horizontal transfer of the OXA-24 carbapenemase gene via outer membrane vesicles: a new mechanism of dissemination of carbapenem resistance genes in *Acinetobacter baumannii*. *Antimicrob. Agents Chemother.* 55 3084–3090. 10.1128/AAC.00929-10 21518847 PMC3122458

[B60] Sabuda D. M. Laupland K. Pitout J. Dalton B. Rabin H. Louie T. (2008). Utilization of colistin for treatment of multidrug-resistant *Pseudomonas aeruginosa*. *Can. J. Infect. Dis. Med. Microbiol.* 19 413–418. 10.1155/2008/743197 19436571 PMC2663472

[B61] Salazar V. A. Arranz-Trullén J. Navarro S. Blanco J. A. Sánchez D. Moussaoui M. (2016). Exploring the mechanisms of action of human secretory RNase 3 and RNase 7 against *Candida albicans*. *Microbiologyopen* 5 830–845. 10.1002/mbo3.373 27277554 PMC5061719

[B62] Sánchez D. Moussaoui M. Carreras E. Torrent M. Nogués V. Boix E. (2011). Mapping the eosinophil cationic protein antimicrobial activity by chemical and enzymatic cleavage. *Biochimie* 93 331–338. 10.1016/j.biochi.2010.10.005 20951760

[B63] Schwechheimer C. Kuehn M. J. (2015). Outer-membrane vesicles from gram-negative bacteria: biogenesis and functions. *Nat. Rev. Microbiol.* 13 605–619. 10.1038/nrmicro3525 26373371 PMC5308417

[B64] Sievers F. Higgins D. G. (2018). Clustal omega for making accurate alignments of many protein sequences. *Protein Sci.* 27 135–145. 10.1002/pro.3290 28884485 PMC5734385

[B65] Sjostrom A. E. Sandblad L. Uhlin B. E. Wai S. N. (2015). Membrane vesicle-mediated release of bacterial RNA. *Sci. Rep.* 5:15329 10.1038/srep15329 PMC4612299 26483327

[B66] Starkey M. Lepine F. Maura D. Bandyopadhaya A. Lesic B. He J. (2014). Identification of anti-virulence compounds that disrupt quorum-sensing regulated acute and persistent pathogenicity. *PLoS Pathog.* 10:e1004321 10.1371/journal.ppat.1004321 PMC4140854 25144274

[B67] Stein A. Raoult D. (2002). Colistin: an antimicrobial for the 21st century? syndrome associated with abacavir therapy of enterococcus faecalis prosthetic valve endocarditis with linezolid. *Clin. Infect. Dis.* 35 901–902. 12228836 10.1086/342570

[B68] Tabbene O. Di Grazia A. Azaiez S. Ben Slimene I. Elkahoui S. Alfeddy M. N. (2015). Synergistic fungicidal activity of the lipopeptide bacillomycin D with amphotericin B against pathogenic candida species. *FEMS Yeast Res.* 15:fov022 10.1093/femsyr/fov022 25956541

[B69] Torrent M. Andreu D. Nogués V. M. Boix E. (2011a). Connecting peptide physicochemical and antimicrobial properties by a rational prediction model. *PLoS One* 6:e16968 10.1371/journal.pone.0016968 PMC3036733 21347392

[B70] Torrent M. Valle J. Nogués M. V. Boix E. Andreu D. (2011b). The generation of antimicrobial peptide activity: a trade-off between charge and aggregation? *Angew. Chem. Int. Ed. Engl.* 50 10686–10689. 10.1002/anie.201103589 21928454

[B71] Torrent M. Cuyás E. Carreras E. Navarro S. López O. De La Maza A. (2007). Topography studies on the membrane interaction mechanism of the eosinophil cationic protein. *Biochemistry* 46 720–733. 10.1021/bi061190e 17223693

[B72] Torrent M. de la Torre B. G. Nogués V. M. Andreu D. Boix E. (2009a). Bactericidal and membrane disruption activities of the eosinophil cationic protein are largely retained in an N-terminal fragment. *Biochem. J.* 421 425–434. 10.1042/BJ20082330 19450231

[B73] Torrent M. Sánchez D. Buzón V. Nogués M. V. Cladera J. Boix E. (2009b). Comparison of the membrane interaction mechanism of two antimicrobial RNases: RNase 3/ECP and RNase 7. *Biochim. Biophys. Acta Biomembr.* 1788 1116–1125. 10.1016/j.bbamem.2009.01.013 19366593

[B74] Torrent M. Navarro S. Moussaoui M. Nogues M. V. Boix E. (2008). Eosinophil cationic protein high-affinity binding to bacteria-wall lipopolysaccharides and peptidoglycans. *Biochemistry* 47 3544–3555. 10.1021/bi702065b 18293932

[B75] Torrent M. Odorizzi F. Nogués M. V. Boix E. Nogues M. V. Boix E. (2010). Eosinophil cationic protein aggregation: identification of an N-terminus amyloid prone region. *Biomacromolecules* 11 1983–1990. 10.1021/bm100334u 20690710

[B76] Torrent M. Pulido D. Nogués M. V. Boix E. (2012). Exploring new biological functions of amyloids: bacteria cell agglutination mediated by host protein aggregation. *PLoS Pathog.* 8:e1003005 10.1371/journal.ppat.1003005 PMC3486885 23133388

[B77] Torrent M. Pulido D. Valle J. Nogués M. V. Andreu D. Boix E. (2013). Ribonucleases as a host-defence family: evidence of evolutionarily conserved antimicrobial activity at the N-terminus. *Biochem. J.* 456 99–108. 10.1042/BJ20130123 23962023

[B78] Vega N. M. Gore J. (2014). Collective antibiotic resistance: mechanisms and implications. *Curr. Opin. Microbiol.* 21 28–34. 10.1016/j.mib.2014.09.003 25271119 PMC4367450

[B79] Webb B. Sali A. (2016). Comparative protein structure modeling using modeller. *Curr. Protoc. Protein Sci.* 54 56.1–5.6.37. 10.1002/cpps.20. PMC5031415 27322406

[B80] Wiegand I. Hilpert K. Hancock R. E. W. (2008). Agar and broth dilution methods to determine the minimal inhibitory concentration (MIC) of antimicrobial substances. *Nat. Protoc.* 3 163–175. 10.1038/nprot.2007.521 18274517

[B81] Wright G. D. (2016). Antibiotic adjuvants: rescuing antibiotics from resistance. *Trends Microbiol.* 24 862–871. 10.1016/j.tim.2016.06.009 27430191

[B82] Yang J. T. Wu C. S. C. Martinez H. M. (1986). Calculation of protein conformation from circular dichroism. *Methods Enzymol.* 130 208–269. 10.1016/0076-6879(86)30013-2 3773734

[B83] Yeaman M. R. (2003). Mechanisms of antimicrobial peptide action and resistance. *Pharmacol. Rev.* 55 27–55. 10.1124/pr.55.1.2 12615953

