# Supplementary figures and images for: Testing a Human Antimicrobial RNase Chimera Against Bacterial Resistance
Source: Front Microbiol. 2019 Jun 19;10:1357. doi: 10.3389/fmicb.2019.01357 (PMC6594349; doi:10.3389/fmicb.2019.01357)

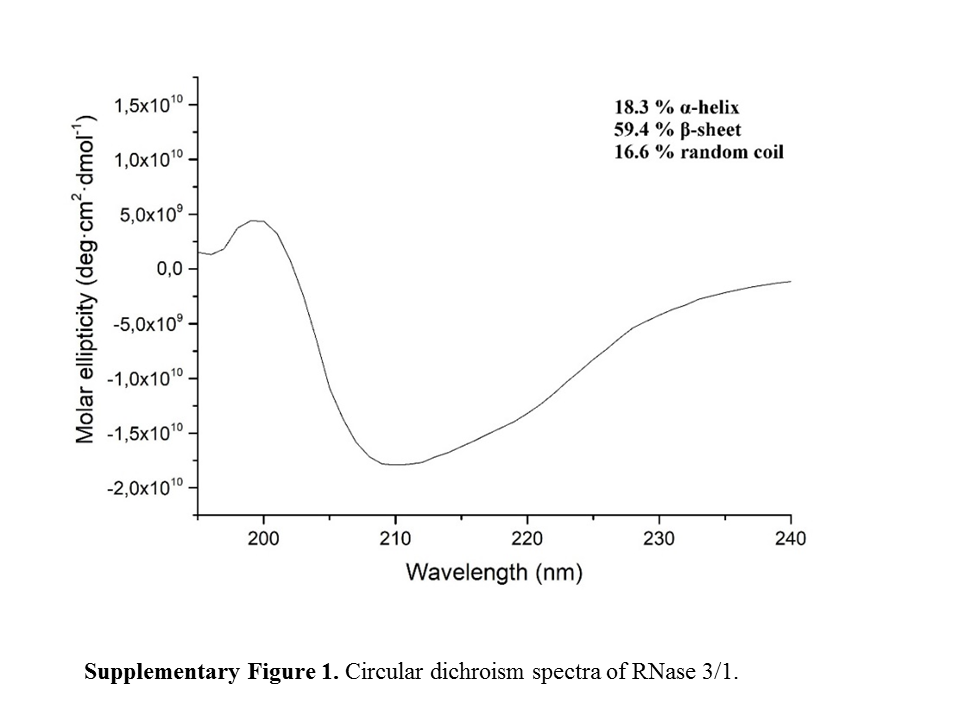

Supplement: Supplementary file 1 [file Image_1.TIF]

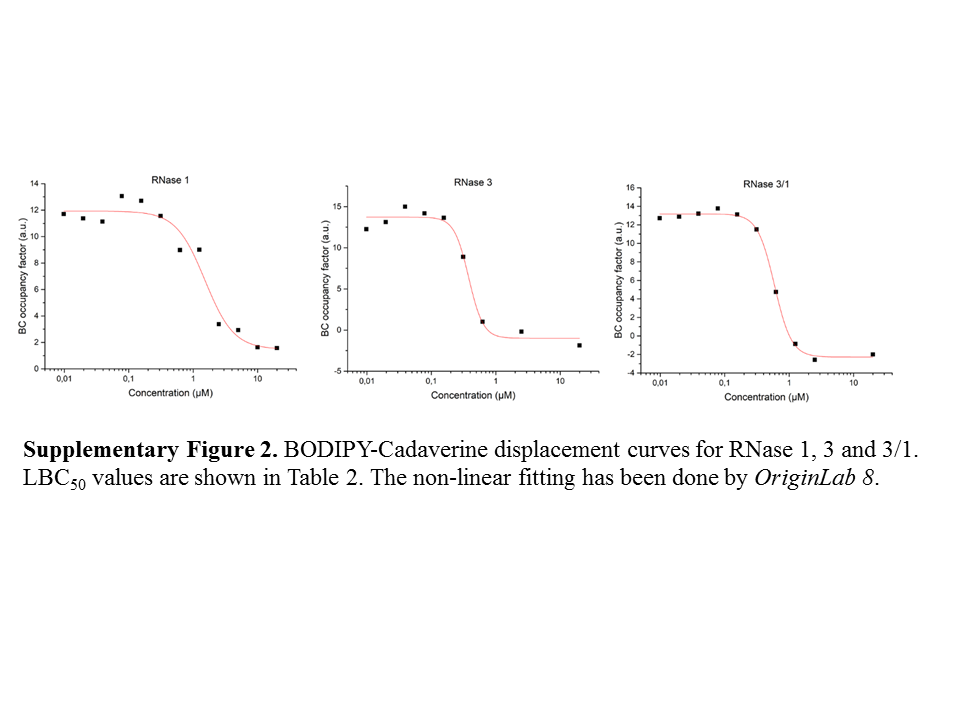

Supplement: Supplementary file 2 [file Image_2.TIF]

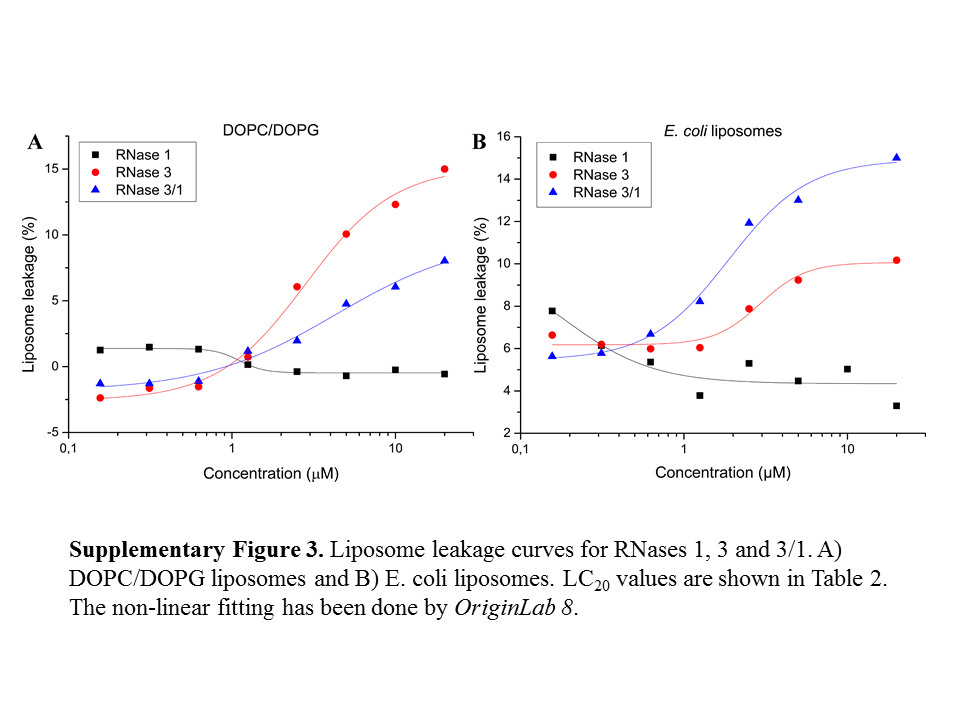

Supplement: Supplementary file 3 [file Image_3.tif]

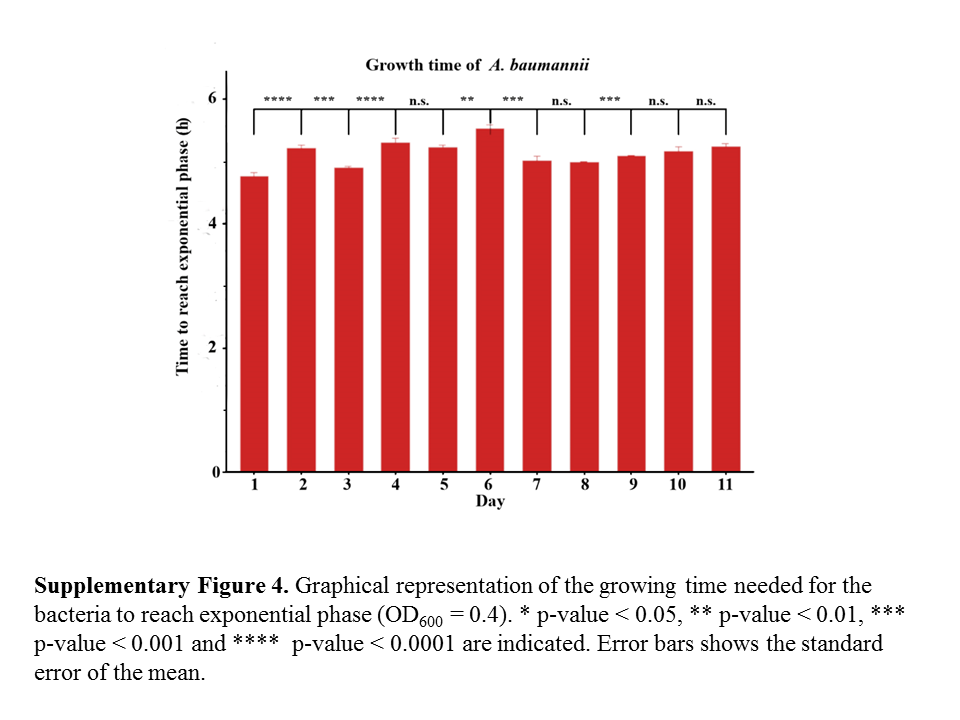

Supplement: Supplementary file 4 [file Image_4.TIF]

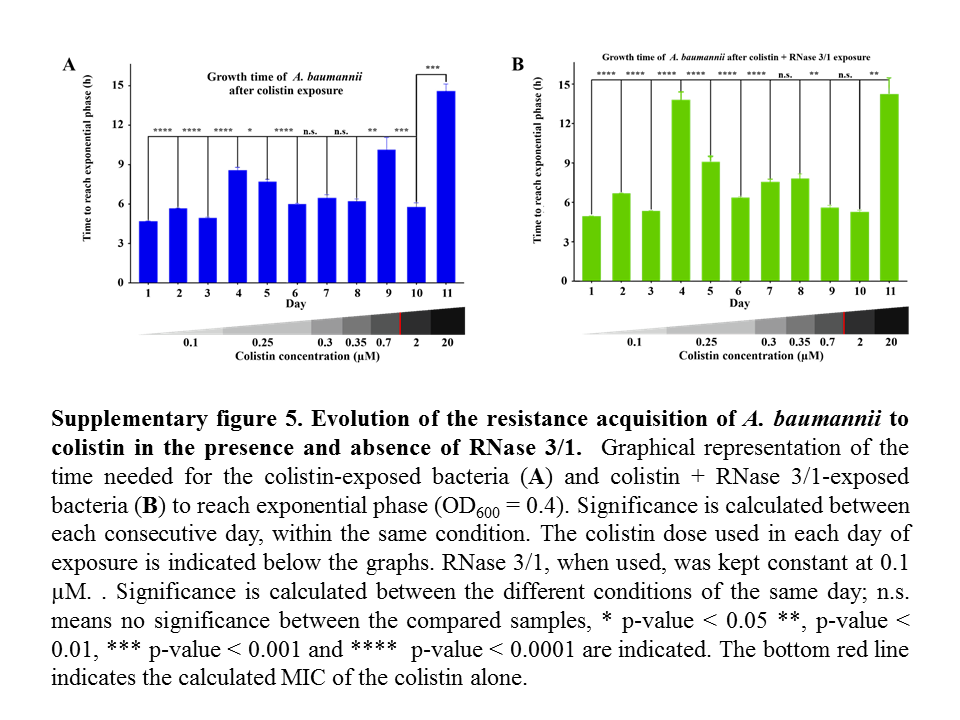

Supplement: Supplementary file 5 [file Image_5.TIF]

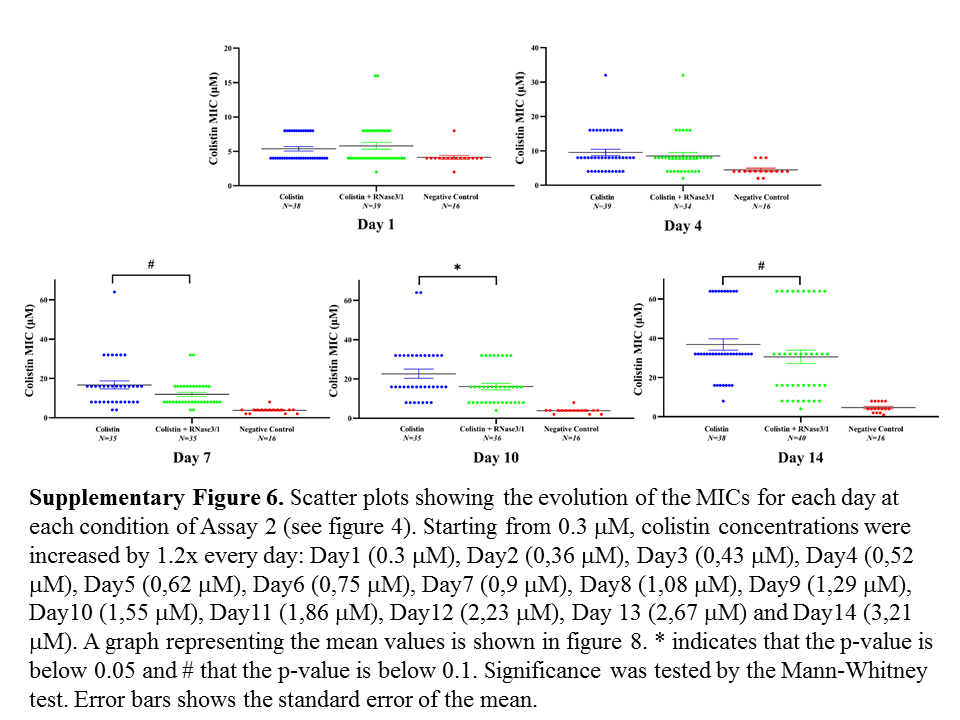

Supplement: Supplementary file 6 [file Image_6.TIF]
